# Observation of van der Waals resonances in low-energy F + H_2_(*v* = 0, *j* = 1) reaction

**DOI:** 10.1093/nsr/nwag086

**Published:** 2026-02-12

**Authors:** Heilong Wang, Wei Wang, Zhirun Jiao, Yu Li, Hongtao Zhang, Bina Fu, Jiayu Huang, Chunlei Xiao, Dong H Zhang, Xueming Yang

**Affiliations:** State Key Laboratory of Chemical Reaction Dynamics, Dalian Institute of Chemical Physics, Chinese Academy of Sciences, Dalian 116023, China; State Key Laboratory of Chemical Reaction Dynamics, Dalian Institute of Chemical Physics, Chinese Academy of Sciences, Dalian 116023, China; School of Chemical Sciences, University of Chinese Academy of Sciences, Beijing 100049, China; State Key Laboratory of Chemical Reaction Dynamics, Dalian Institute of Chemical Physics, Chinese Academy of Sciences, Dalian 116023, China; School of Chemical Sciences, University of Chinese Academy of Sciences, Beijing 100049, China; State Key Laboratory of Chemical Reaction Dynamics, Dalian Institute of Chemical Physics, Chinese Academy of Sciences, Dalian 116023, China; School of Chemical Sciences, University of Chinese Academy of Sciences, Beijing 100049, China; State Key Laboratory of Chemical Reaction Dynamics, Dalian Institute of Chemical Physics, Chinese Academy of Sciences, Dalian 116023, China; State Key Laboratory of Chemical Reaction Dynamics, Dalian Institute of Chemical Physics, Chinese Academy of Sciences, Dalian 116023, China; Hefei National Laboratory, Hefei 230088, China; State Key Laboratory of Chemical Reaction Dynamics, Dalian Institute of Chemical Physics, Chinese Academy of Sciences, Dalian 116023, China; Key Laboratory of Materials Modification by Laser, Ion and Electron Beams (Dalian University of Technology), Ministry of Education, Dalian 116024, China; State Key Laboratory of Chemical Reaction Dynamics, Dalian Institute of Chemical Physics, Chinese Academy of Sciences, Dalian 116023, China; Hefei National Laboratory, Hefei 230088, China; State Key Laboratory of Chemical Reaction Dynamics, Dalian Institute of Chemical Physics, Chinese Academy of Sciences, Dalian 116023, China; Hefei National Laboratory, Hefei 230088, China; State Key Laboratory of Chemical Reaction Dynamics, Dalian Institute of Chemical Physics, Chinese Academy of Sciences, Dalian 116023, China; Hefei National Laboratory, Hefei 230088, China; Department of Chemistry & Shenzhen Key Laboratory of Energy Chemistry, Southern University of Science and Technology, Shenzhen 518055, China

**Keywords:** crossed molecular beam, vdW resonance state, entrance-channel resonances, spin-orbit coupling, low-temperature chemistry, reaction dynamics

## Abstract

Quantum resonances exert a crucial impact on chemical reactivity, but experimental evidence has so far been restricted to transition-state or product-channel features. Here we provide the first direct evidence of van der Waals (vdW) resonances in the entrance channel of a neutral molecular reaction. Using fully quantum-state resolved crossed molecular beam scattering, we studied the benchmark F + H_2_(*v* = 0, *j* = 1) reaction over collision energies from 4.0 to 26.4 cm^−1^. A pronounced forward scattering peak of the HF(*v*′ = 2) product at 6.8 cm^−1^ reveals a resonance arising from quasi-bound states trapped in the entrance-channel vdW well, facilitated by centrifugal barriers. This signature is reproduced by quantum dynamical calculations on an open-shell diabatic potential energy surface, revealing partial wave resonances with total angular momentum (*J*) = 6.5–7.5 and demonstrating that spin-orbit coupling decisively shifts the energies of these quasi-bound states, shaping both the resonance position and scattering distribution. This combined experimental and theoretical study establishes a general quantum mechanism likely to influence a wide range of elementary reactions at low temperatures, including those relevant to interstellar chemistry and cold controlled systems.

## Teaser

Entrance-channel van der Waals resonances are directly observed for the first time, and shown to shape chemical reactivity at low collision energies in a neutral molecular reaction.

## INTRODUCTION

A reaction resonance is a quasi-bound quantum state trapped transiently in a potential well along the reaction coordinate in the transition state region of a chemical reaction [1–3]. When the energy of a reaction system matches a reaction resonance, the outcome of the reaction, including the reaction probability, angular distribution, and quantum state distribution of the products may change rapidly. These rapid variations of chemical reactivity are extremely sensitive to the details of the potential well that confines the resonance state. Therefore, the observation and characterization of reaction resonances is not only important for understanding the quantum nature of the transition state, but also a very sensitive probe for the potential energy surface (PES) in the transition state region [4,5].

Reaction resonance has been a fascinating research subject since its theoretical conception in the 1970s [6–8]. Early evidence was found in crossed beam experiments of the F + H_2_ reaction and its isotope variants [9–14]. Subsequently, more detailed studies have been carried out on this benchmark system using high-resolution crossed molecular beam scattering experiments as well as negative ion photoelectron spectroscopy experiments [15–18]. The reaction resonances observed in the F + H_2_ reaction and its isotope variants were identified as resonances trapped in the vibrationally adiabatic potential in the exit channel of this reaction. The intriguing role of these resonances in the formation of HF molecules in interstellar clouds has also been elucidated [19,20]. In addition to the F + H_2_ system, resonances in the exit channel have been investigated in other chemical reactions, such as F + H_2_O [21,22], F + NH_3_ [23,24], and F + CH_3_OH [25], etc. Recently, reaction resonances were also observed in Cl + HD(*v* = 1) → HCl + D near its reaction barrier as a result of a chemical bond-softening mechanism [26]. This mechanism is expected to exist in many reactions involving vibrationally excited molecules [22,27]. Thus, reaction resonances in chemical reactions have been detected either near the reaction barrier or in the exit channel.

Partial wave resonances have been detected in inelastic scattering [28,29] and Penning ionization processes [30,31] in the entrance van der Waals (vdW) wells. Given the ubiquitous nature of vdW interactions and centrifugal potentials in atomic and molecular collisions, it is theoretically possible to form a potential well that is capable of supporting shape resonance states in the vdW well region in all chemical reactions, as in the inelastic scattering or Penning ionization processes.

There are also some discussions about quantum reactive resonances due to vdW wells in the entrance. For example, quantum dynamics calculations for O(^3^P) + HCl revealed pronounced resonance features attributed to quasi-bound states supported by vdW wells in both the entrance and exit channels [32]. In addition, for F + CH_4_, vdW resonance structures were theoretically characterized and revealed via transition-state spectroscopy of FCH_4_ [33]. The existence of similar resonances in the F + H_2_ reaction and its isotopic variants has previously been proposed [18,34–36]. These studies suggest that the reaction dynamics could be substantially affected by these resonances. However, direct experimental observation of entrance-channel vdW resonances under reactive scattering conditions, as well as their impact on chemical reactivity, remains elusive to date, possibly due to the fact that these resonances could be detected only at very low collision energies that are not readily accessible. Because of the likely significance of these resonances in chemical reactions, it is highly desirable to observe and understand them with high precision. In this work, we investigate the role of entrance-channel vdW interactions in reactive scattering of the F + H_2_ (*v* = 0, *j* = 1) system at low collision energies using high-resolution crossed molecular beam experiments in combination with rigorous quantum dynamical calculations. By accessing a previously unexplored energy regime, this study aims to elucidate whether quasi-bound states supported by the entrance-channel vdW well can influence chemical reactivity under fully reactive scattering conditions.

## RESULTS AND DISCUSSION

In this study, we carried out a fully quantum-state-resolved, crossed beam scattering experiment on the F(^2^P_3/2_) + H_2_(*v* = 0, *j* = 1) → HF(*v*′, *j*′) + H reaction in a low collision energy range between 4.0 and 26.4 cm^−1^, using the high-resolution and high-sensitivity H-atom Rydberg tagging method. The experimental apparatus for this experiment has been described in detail [37]. Both the fluorine atom beam and the H_2_ molecule beam were generated via supersonic expansion from pulsed valves that were cooled down with liquid nitrogen to reduce the beam velocities as well as the absolute velocity spreads, in order to reduce the collision energy and improve the experimental resolution. The two reactant beams were collimated by skimmers and then entered a scattering chamber where they intersected with each other at the crossing region. To achieve low collision energy, the crossing angle between the two beams may be varied, with a minimum of 15° achievable in this apparatus; while the velocities of both beams were also tunable by changing the ratio of different carrier gases. The H atoms produced were first excited via a two-step excitation scheme to a high Rydberg state in the crossing region, and these Rydberg tagged H atoms then flew ∼256 mm before reaching a micro-channel plate (MCP) detector with a fine metal mesh in front of the MCP. After passing through the mesh, the Rydberg H atoms were immediately field-ionized and detected by the MCP detector. The signals collected by the MCP detector were amplified, discriminated, and then recorded in the form of the time-of-flight (TOF) spectrum. To obtain the TOF spectra for the F + H_2_(*v* = 0, *j* = 1) reaction, experiments were performed for both *n*-H_2_ (*normal*-H_2_) and *p*-H_2_ (*para*-H_2_) reactions under the exact same experimental conditions. In the cold molecular beam condition, the *n*-H_2_ beam has 25% H_2_(*j* = 0) and 75% H_2_(*j* = 1), while the *p*-H_2_ beam has 100% H_2_(*j* = 0). From these measurements, the TOF spectra from the H_2_(*j* = 1) reaction can be obtained by subtracting the H_2_(*j* = 0) reaction contribution from the *n*-H_2_ reaction signals with the correct relative percentage (see section II in the Supplementary Materials for a more detailed description of the data treatment).

**Figure 1.**
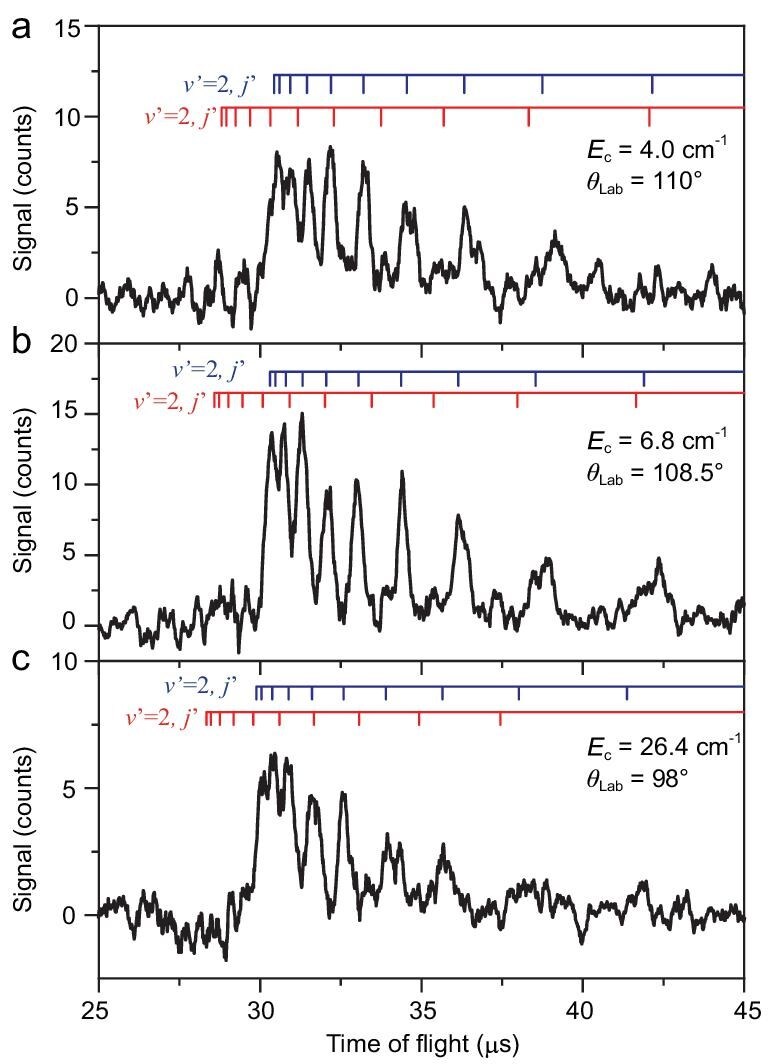
Time-of-flight (TOF) spectra of the H atom product from the F + H_2_(*v* = 0, *j* = 1) → HF + H reaction in the forward scattering direction in the center-of-mass frame. They were obtained at three collision energies (*E*_c_): 4.0 cm^−1^ (a), 6.8 cm^−1^ (b), and 26.4 cm^−1^ (c), respectively, in roughly the forward scattering direction in the center-of-mass frame. The peaks can be assigned to the rovibrational states of the HF(*v*′ = 2, *j*′) product from F(^2^P_3/2_) + H_2_(*v* = 0, *j* = 1) → HF(*v*′ = 2, *j*′) + H (blue lines) and F*(^2^P_1/2_) + H_2_(*v* = 0, *j* = 1) → HF(*v*′ = 2, *j*′) + H (red lines), respectively.

TOF spectra of the H atom products from the F + H_2_(*v* = 0, *j* = 1) → HF(*v*′, *j*′) + H reaction were acquired at collision energies ranging from 4.0 to 26.4 cm^−1^. Figure 1 shows three typical TOF spectra in the forward scattering direction at collision energies of 4.0, 6.8, and 26.4 cm^−1^, respectively. The main structures in the TOF spectra can be assigned to a series of rotational states of the HF(*v*′ = 2) product from the F-atom spin-orbit ground state reaction, F(^2^P_3/2_) + H_2_. There are also some smaller peaks in the TOF spectra that could be assigned to the HF(*v*′ = 2) product from the spin-orbit excited F-atom reaction, F(^2^P_1/2_) + H_2_; HF products populating other vibrational states are negligible. For direct comparison, all the TOF spectra have been normalized, as described in the Supplementary Materials. From these TOF spectra, the differential cross section (DCS) for the HF(*v*′ = 2) product from the F(^2^P_3/2_) + H_2_ reaction in the forward scattering direction can thus be determined at collision energies from 4.0 to 26.4 cm^−1^.

**Figure 2.**
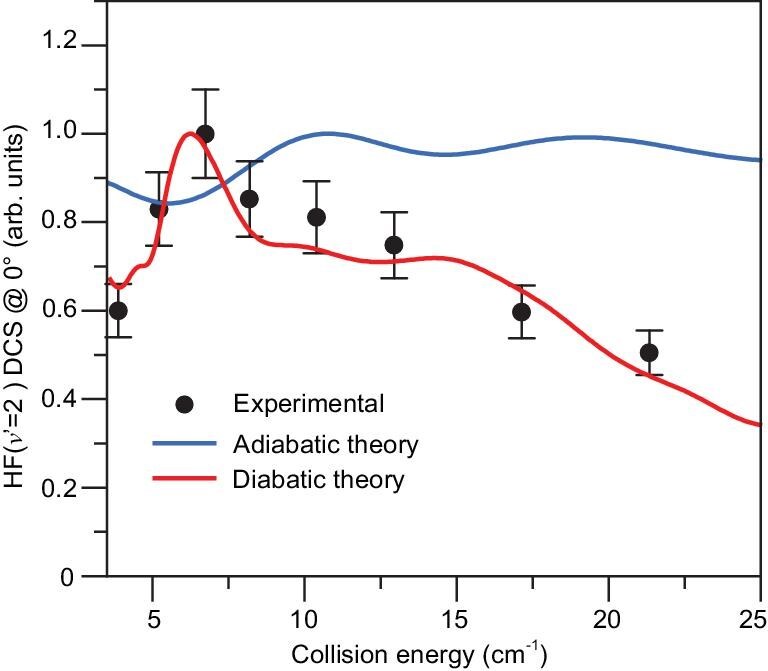
Collision-energy–dependent differential cross sections in the forward scattering direction for the F(^2^P_1/2_) + H_2_(*v* = 0, *j* = 1) → HF(*v*′ = 2, *j*′) + H reaction in the low collision energy regime from 4.0 to 26.4 cm^−1^. The black solid circles (these data are obtained from the analysis of raw TOF spectra exemplified in Fig. 1) represent the experimental QSSFSS for the HF (*v*′ = 2) product from the F + H_2_(*v* = 0, *j* = 1) reaction. The solid red line is the corresponding calculated QSSFSS using the diabatic theory including spin-orbit couplings. The solid blue line is the result of adiabatic theory. Analysis of experimental data and evaluation of error bars (1*σ*) are discussed in the Supplementary Materials.

**Figure 3.**
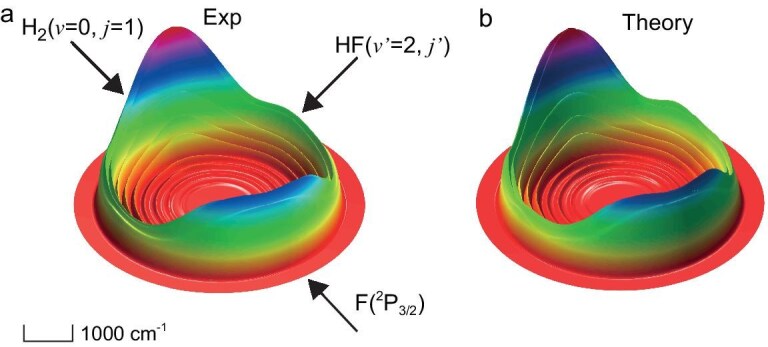
Experimental (a) and theoretical (b) three-dimensional scattering contour plots for HF(*v*′ = 2, *j*′) products from the reaction of F + H_2_(*v* = 0, *j* = 1) → HF(*v*′ = 2, *j*′) + H at the collision energy corresponding to peak location in the QSSFSS (6.8 cm^−1^ for experiment and 6.45 cm^−1^ for theory). The forward-scattering direction for HF (*v*′ = 2) is defined along the direction of the F atom beam. The recoil energy scale is represented by the bar located in the bottom right corner of this figure.

Figure 2 shows the experimentally measured DCS for the HF(*v*′ = 2) product from this reaction in the forward scattering direction at several collision energies. The forward scattering DCS signal for the HF(*v*′ = 2) product first increases and then decreases as the collision energy increases, exhibiting a distinct maximum at 6.8 cm^−1^ collision energy. This collision-energy–dependent forward scattering peak has never been reported in this energy region before, and it is substantially below the excited resonance (23.4 cm^−1^) in the exit channel of this reaction. In addition, angular-resolved TOF spectra were also measured for the F(^2^P_3/2_) + H_2_(*v* = 0, *j* = 1) → HF(*v*′ = 2) + H reaction at 6.8 cm^−1^ collision energy. The experimental angular-resolved DCS for HF(*v*′ = 2) is shown in Fig. 3a. The DCS exhibits large forward and backward scattering features for the HF(*v*′ = 2) product. The energy-dependent DCS peak in the forward scattering direction is particularly interesting because it seems to be not associated with any resonance in the exit channel that has been well characterized previously. Furthermore, the angular-resolved DCS shows a strong forward scattering peak with significant backward scattering signal, which is characteristic of reaction resonances.

To interpret these interesting experimental observations, we first carried out adiabatic calculations on the CSZ PES, using the time-independent ABC code [38]. This PES has been proven to be accurate enough to reproduce the dynamics of the F + H_2_ reaction as well as its isotopic analogues in a broad range of collision energies [16,39]. The parameters adopted in the calculation are given in the Supplementary Materials. Our time-independent adiabatic calculation reveals two sharp peaks in the reaction probability of total angular momentum *J* = 6 and 7 partial waves (see Fig. S3) in the collision energy range investigated in this work, suggesting the presence of previously undiscovered reaction resonances in the adiabatic picture.

We computed the collision-energy–dependent differential cross sections for a specific product quantum state in the forward scattering direction, designated as the quantum-state-specific forward scattering spectroscopy (QSSFSS), for the HF(*v*′ = 2) product and compared it with the experimental results in Fig. 2. It is important to note that the collision energy broadening in the experiment varies with collision energy. Therefore, a careful Gaussian convolution of the theoretically calculated results was performed using the experimentally estimated collision energy broadening (Table S2) to compare with the experimental QSSFSS, as explained in the Supplementary Materials. The unbroadened theoretical results are shown in Fig. S5. The adiabatic QSSFSS only shows slight oscillatory structures that are completely different from the experimental result, indicating that the quantum dynamics calculations based on the adiabatic model fail to reproduce the experimentally observed peak in the QSSFSS.

To clarify whether this large discrepancy between the theoretical and experimental QSSFSS was caused by the simplified adiabatic model, which treats the F atom as a structureless entity and neglects its electron spin and orbit motion, we carried out full diabatic time-dependent wave packet calculations for the F(^2^P_3/2_) + H_2_(*v* = 0, *j* = 1) reaction based on the diabatic iCSZ-LWAL PESs that include full spin and orbit motions of the F-atom and all relevant couplings. The iCSZ-LWAL PES was constructed from the iCSZ PES and the diabatic LWAL PESs (for details, see Supplementary Information of Ref. 20). In this work, we further refined the diabatic coupling elements in the LWAL PES, resulting in an improved iCSZ-iLWAL PES with an energy error reduced to <0.2 cm^−1^. This represents a substantial improvement over the previous version, which had an error exceeding 10 cm^−1^. Comparison between the forward-scattering DCSs from the adiabatic and diabatic models reveals that spin-orbit splitting causes a substantial shift of the peak positions in the diabatic calculations towards low energy, particularly in the low collision energy regime. The theoretical QSSFSS convoluted with experimental collision energy broadening (Fig. 2) and DCS (Fig. 3b) are now in excellent agreement with the experimental result, when full open-shell characteristics of the F atom were considered, indicating that the fine structures caused by the couplings between electron spin, orbital angular momentum, and nuclear rotational angular momentum play a crucial role in the dynamics of the F(^2^P_3/2_) + H_2_(*j* = 1) reaction in this low collision energy regime. Figure S6 presents the integral cross sections (ICSs) and partial wave contributions from the adiabatic and diabatic calculations at low collision energies. Compared with the adiabatic results (Fig. S6a), the resonance peaks are less pronounced in the diabatic calculations. In the diabatic case, partial wave contributions from orbital angular momentum *L* (Fig. S6b) exhibit clear resonances for *L* = 4–6, while the resonance at *L* = 6, although smaller in total weight, displays a more pronounced peak structure. The contributions from total angular momentum *J* (Fig. S6c) show distinct resonances at *J* = 5.5, 6.5, and 7.5, with the peak at *J* = 6.5 dominating.

We then performed a detailed dynamics analysis for the forward scattering DCS to understand the nature of the resonances. The behavior of spin-orbit split partial waves in the full open-shell model deserves more explanation. Unlike the adiabatic picture, the diabatic model fully incorporates electron spin and orbital angular momentum into the calculations. Consequently, the total angular momentum *J* is the sum of the nuclear orbital angular momentum *L* and the internal angular momentum *j*_12_, where *j*_12_ is the sum of the diatomic angular momentum *j*, and the electronic angular momentum *j_a_*. The electronic angular momentum *j_a_* is the sum of electron spin *s* and orbital angular momentum *l*, following Hund’s case (a). In the specific case of the F(^2^P_3/2_) + H_2_(*j* = 1) reaction, the rotational motion of the H_2_ molecule allows for three possible values for *k*: −1, 0, and 1. Here, *k* represents the projection of the angular momentum onto the chosen quantization axis (for details, see section V in the Supplementary Materials). Meanwhile, the initial ^2^P_3/2_ state of fluorine has *s* = 1/2 and *l* = 1, allowing *k_a_*, the projection of *j_a_*, to take four values: 1.5, 0.5, −0.5, and −1.5. The variability in both *k* and *k_a_* leads to 12 partial waves for a single total angular momentum number *J* in the diabatic model.

Figure S7a shows the forward-scattering DCS calculated using the full open-shell diabatic model without convolution of experimental broadening. It is evident that the experimental peak shown in Fig. 2 is contributed by the prominent resonance peak at 6.45 cm^−1^. This shift explains the large differences observed between the QSSFSS calculations for the HF(*v*′ = 2) product when using the adiabatic model versus the full spin-orbit coupled model. Figure S7b shows the relative reaction probabilities as a function of collision energy for four spin-orbit fine-structure partial waves with the most prominent resonance peaks. As can be seen, the (*J* = 6.5, *L* = 4, *j*_12_ = 2.5, parity = +1, labeled as PW-L4) and (*J* = 6.5, *L* = 6, *j*_12_ = 1.5, parity = +1, labeled as PW-L6) partial waves are the primary contributors to the forward scattering peak at 6.45 cm^−1^. The energy widths of the individual partial wave resonance peaks are ∼1 cm^−1^, which corresponds to a lifetime of about 6 picoseconds, substantially longer than the lifetime of the resonance state (∼100 fs) in the exit channel in the reaction.

**Figure 4.**
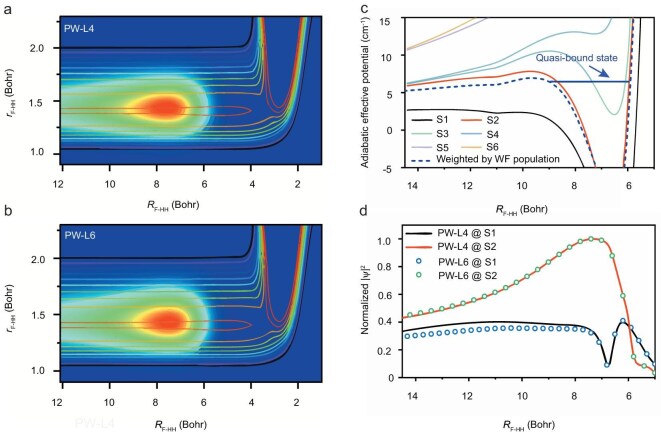
One-dimensional time-independent scattering wave functions and adiabatic effective potentials for the F(^2^P_3/2_) + H_2_(*v* = 0, *j* = 1) → HF(*v*′ = 2, *j*′) + H reaction. (a and b) Scattering wave functions for partial waves (*J* = 6.5, *L* = 4, *j*_12_ = 2.5, parity = +1, labeled as PW-L4) and (*J* = 6.5, *L* = 6, *j*_12_ = 1.5, parity = +1, labeled as PW-L6) at a collision energy of *E*_c_ = 6.45 cm^−1^. (c) One-dimensional adiabatic effective potentials along the *R*_F-HH_ direction, calculated by averaging over the vibrational dimension and diagonalizing the matrix representation of the potential energy over the angular dimension. For the F(^2^P_3/2_) + H_2_(*j* = 1) reaction with fixed quantum numbers *J* = 6.5 and parity = +1, diagonalization over the angular basis yields six adiabatic channel eigenstates in the asymptotic region. The corresponding one-dimensional adiabatic effective potentials are labeled S1–S6. S1–S6 therefore denote the six asymptotic incident-channel eigenstates (and their associated adiabatic effective potentials). The labels are ordered by the asymptotic eigenvalues/threshold energies. The quasi-bound state label denotes the underlying entrance-channel shape resonance state coupled to both PW-L4 and PW-L6, which produces two resonance peaks in the partial-wave reaction probabilities. The resonance features are governed primarily by S1 and S2, with S3 contributing only weakly. (d) Time-independent scattering wave functions populated in the S1 and S2 states are shown for both PW-L4 and PW-L6.

To uncover the dynamic origins of these resonance peaks, we extracted the scattering resonance wave functions (WFs) for the PW-L4 and PW-L6 partial waves from our time-dependent wave packet calculations. Figure 4a and b illustrate the two-dimensional resonance WFs as a function of the *R* (the distance between F and H_2_) and *r* (the bond length of H_2_), with the third coordinate *θ* integrated. The resonance WFs for PW-L4 and PW-L6 are essentially identical, located in the entrance channel for the reaction with very long tails, totally different from the Feshbach resonances in the exit channel discovered in the reaction. In addition, there are no nodes in the *R* and *r* coordinates of the WF, and the shape of the WF in the *θ* coordinates is very similar to the shape of the associated Legendre polynomials *P*_1,1_(*θ*), as the wavefunction is predominantly populated in the (*j* = 1, *k* = 1) state. Therefore, these resonance states are shape resonances trapped in the vdW potential well region in the entrance channel.

The dynamic origin of the shape resonances is attributed to the combined effects of vdW and centrifugal interactions. For orbital angular momentum *L* = 0, the attractive vdW interactions and repulsive forces from the reaction barrier form a relatively wide vdW well in the entrance channel. With the increase of *L*, the centrifugal potential raises the vdW well and forms a centrifugal barrier outside the vdW well, making it possible to support some shape resonance states, as can be seen clearly from the adiabatic calculations, in which partial waves with *J* = 6 and 7 show strong resonance peaks at collision energies of 6.6 cm^−1^ and 8.7 cm^−1^, respectively (for details, see section V in the Supplementary Materials). In the full open-shell model, the inclusion of spin-orbit couplings leads to the splitting of partial waves, resulting in a more complex picture of reaction dynamics. For the F(^2^P_3/2_) + H_2_(*j* = 1) reaction with fixed quantum numbers *J* = 6.5 and parity = +1, there are a total of six degenerate incident states in the asymptotic region: (*L* = 4, *j*_12_ = 2.5), (*L* = 6, *j*_12_ = 0.5, 1.5, 2.5), and (*L* = 8, *j*_12_ = 1.5, 2.5). Among these, the (*L* = 4, *j*_12_ = 2.5) and (*L* = 6, *j*_12_ = 1.5) initial states are of particular importance, since their reaction probabilities show prominent resonance peaks at 6.45 cm^−1^ (Fig. S6b). As H_2_ approaches the F atom, these six states become coupled due to spin-orbit and non-adiabatic interactions, as well as the centrifugal potential, gradually splitting into six distinct adiabatic states. These states are labeled S1 to S6 in the order of increasing energy. The one-dimensional effective potentials corresponding to these adiabatic incident states (solid lines in Fig. 4c) can be obtained by diagonalizing the matrix representation of the combined potential energy and centrifugal interaction (for details, see section V in the Supplementary Materials). Out of these six states, the S1, S2, and S3 states possess wells in the 6 Bohr < *R* < 8 Bohr region with centrifugal barriers beyond this range, suggesting the possibility of supporting resonance states.

To identify which adiabatic state(s) support the resonances shown in Fig. 4a and b, we project the resonance WFs to the S1–S6 states. Both resonance WFs for PW-L4 and PW-L6 are predominantly distributed on the S1 and S2 states (Fig. 4d and Fig. S7). The projection coefficients of the resonance WFs on the S1 and S2 states are essentially identical, in particular on the S2 states which have a larger component and exhibit a peak at *R* = 7.4 Bohr. Therefore, although two resonance peaks are observed in the partial wave reaction probabilities of PW-L4 and PW-L6, they arise from the same underlying entrance-channel quasi-bound state trapped in the vdW well. By averaging the S1 and S2 potentials using the projection coefficients shown in Fig. 4d as weights, we obtain an effective adiabatic potential that supports the resonance state (represented by the blue dashed line in Fig. 4d). As can be seen, the centrifugal barrier on the effective potential is only slightly higher than the resonance energy, making the resonance state easily accessible by both PW-L4 and PW-L6.

**Figure 5.**
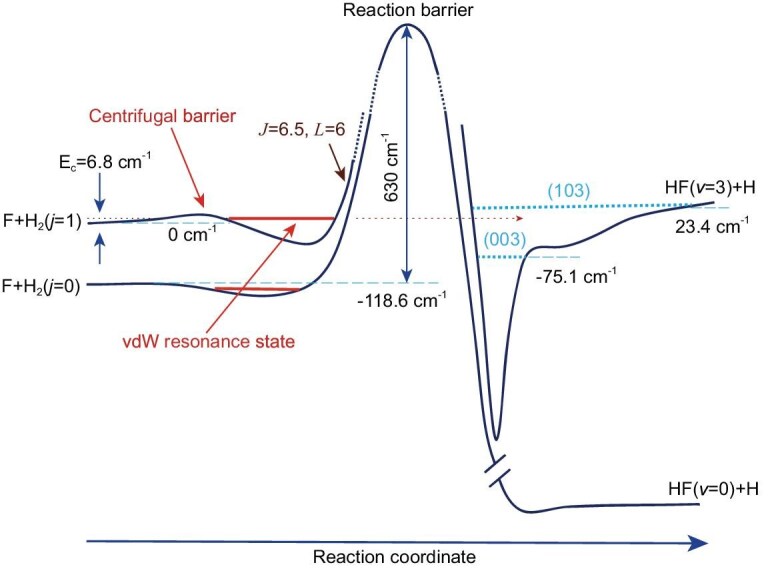
Schematic picture of the reaction resonances at both the entrance channel and the exit channel. In the entrance channel, a typical vdW spin-orbit split partial wave resonance state supported by the centrifugal barrier is shown. The observed forward scattering peak at *E*_c_ = 6.8 cm^−1^ is due to the vdW resonances at the entrance channel. The (003) and (103) resonance states are the ground and first excited resonance states in the exit channel, which were investigated previously.

Therefore, this combined experimental and theoretical study uncovered the existence of vdW resonances in the entrance channel of the F + H_2_(*v* = 0, *j* = 1) reaction at the collision energy equivalent of ∼10 K. The resonance-mediated reaction mechanism for the F + H_2_(*v* = 0, *j* = 1) reaction is illustrated in Fig. 5. Notably, the attractive vdW interactions are critical for the formation of this shape resonance state, as centrifugal forces alone would not create a potential well without the vdW interactions. To reproduce the resonance peak observed in the experiment, accurate diabatic PESs including spin-orbit coupling are necessary in the full diabatic quantum calculations, indicating the importance of spin-orbit interactions in the accurate quantum dynamics of resonances in this reaction. Because the centrifugal barrier and vdW well always exist in the entrance channel of all chemical reactions, similar vdW resonances likely exist in many other chemical reactions at low collision energies. These resonances may not only play important roles in chemical reactions at low temperatures, but also provide an extremely sensitive probe to the details of PES in the entrance valley, and may thus be relevant to the interpretation of low-temperature rate constant measurements [19].

With the reactive vdW resonances observed clearly in the entrance channel in the present study, resonances have now been found in the entire transition state region, i.e. before the reaction barrier, on the barrier as well as after the barrier, suggesting their prevalence in chemical reactions. As a result, considering only the transition state at the barrier is often insufficient for accurately describing chemical reactivity in many reactions, especially in the low collision energy regime. One would need to precisely characterize the entire transition state region, which encompasses long-range interactions both before and after the barrier, where quantum resonances may have a prominent effect on chemical reactivity.

## CONCLUSION

The present results reveal entrance-channel vdW resonances in a neutral molecular reaction for the first time. For the benchmark F + H_2_(*v* = 0, *j* = 1) reaction at low collision energies, crossed molecular beam experiments observe pronounced resonance features manifested as forward-scattering peaks in the collision-energy–dependent differential cross sections of the HF(*v*′ = 2) product. Quantum dynamical calculations reproduce these features and further show that they originate from quasi-bound states supported by the entrance-channel vdW well, facilitated by centrifugal barriers. Because long-range vdW interactions and centrifugal barriers are common features of entrance channels, similar vdW resonances are expected to occur in many other chemical reactions at low collision energies. Our results establish entrance-channel vdW resonances as a general class of quantum reactive resonances and highlight the entrance channel as a key region of chemical reactivity, where long-range interactions and quantum resonances play a decisive role in shaping reaction dynamics.

## Supplementary Material

nwag086_Supplemental_File

## Data Availability

All data needed to evaluate the conclusions in this paper are present in the paper or in the Supplementary Materials.
