# Supplementary material for: Observation of van der Waals resonances in low-energy F + H2(v = 0, j = 1) reaction
Source: Natl Sci Rev. 2026 Feb 12;13(8):nwag086. doi: 10.1093/nsr/nwag086 (PMC13137987; doi:10.1093/nsr/nwag086)
Supplement: nwag086_Supplemental_File [file nwag086_supplemental_file.pdf]

Supplementary Materials for

Observation of van der Waals Resonances in low-energy F + $H_2(v=0, j=1)$ Reactions

Heilong Wang [1),*], Wei Wang [1),4),*], Zhirun Jiao [1),4)], Yu Li [1),4)], Hongtao Zhang [1)], Bina Fu [1),5)], Jiayu Huang [1),3),†], Chunlei Xiao [1),5),†], Dong H. Zhang [1),5),†], Xueming Yang [1),2),5),†]

1) State Key Laboratory of Molecular Reaction Dynamics, Dalian Institute of Chemical Physics, Chinese Academy of Sciences, Dalian, Liaoning 116023, China
2) Department of Chemistry & Shenzhen Key Laboratory of Energy Chemistry, Southern University of Science and Technology, Shenzhen 518055, China
3) Key Laboratory of Materials Modification by Laser, Ion and Electron Beams (Dalian University of Technology), Ministry of Education, Dalian 116024, China
4) School of Chemical Sciences, University of Chinese Academy of Sciences, Beijing, 100049, China
5) Hefei National Laboratory, Hefei, 230088, China

* These authors contributed equally to this work.

† Corresponding author. E-mail: jyhuang@dlut.edu.cn (J.H.); chunleixiao@dicp.ac.cn (C.X.); zhangdh@dicp.ac.cn (D.H.Z.); xmyang@dicp.ac.cn (X.Y.)

This PDF file includes:

Materials and Methods
Figs. S1 to S9
Table S1 to S5

Materials and Methods

I. Experimental details

The experimental apparatus with multi-channel H atom Rydberg tagging time-of-flight detection technique used in the molecular crossed beam scattering study of F + H_2(v=0, j=1) → HF + H reaction has been described previously [37]. The arrangement of the experimental apparatus is shown in Supplementary Figure 1. The SC-I, SC-II, and DA are source chamber I, source chamber II and 15 microchannel plate (MCP) detector assembly, respectively. The F atom beam source is fixed in the source chamber I, while the H_2 molecular beam source inside the source chamber II is rotatable around the beam crossing center. The two molecular beams entered the scattering chamber via skimmers (Beam Dynamics Inc., Model 7, with an opening diameter of 2.0 mm) connecting the source chambers and the scattering chamber and intersect with each other at the crossing region. The crossing angle of the two molecular beams can be varied from 15° and 120°. Thus, the collision energy of the reaction can be varied by changing both the crossing angle and the speed of the two molecular beams.

The mixture of F_2 in He or Ne was expanded through a copper ring sealed pulsed valve (Parker Hannifin Corp., General Valve, Series 9) at a stagnation pressure of 200 psi. The F atoms in the beam were generated either by a double-stage discharge [40] or 248-nm photolysis of this mixture [41]. For the QSSFSS measurement, the double-stage discharge method was used in the experiment; while for angular DCS measurement at 6.8 cm^{-1}, photolysis of F_2 was performed using the 248 nm KrF laser with a pulse energy of 60 mJ, which was produced by an excimer laser (Coherent Inc., COMPex, KrF). The focused 248 nm laser intersected the 5% F_2 /95% Ne beam at the nozzle exit with a spot size of 1mm x 3 mm to produce the F-atom beam. The H_2 beam was generated by expanding the H_2 gas through a pulsed valve (Even-Lavie valve) at a stagnation pressure

of 200 psi, which was cooled down by liquid nitrogen (~ 78 K). The cooling of the nozzle ensured that nearly all *normal*-H_2 molecules are in the j=0 and j=1 states with a ratio of 25% and 75%. The *para*-H_2 sample was made by passing the normal H_2 gas through a ferromagnetic catalyst at ~20 K then stored in an aluminum tank at the room temperature, nearly all H_2 molecules were populated in the j=0 state after supersonic expansion. By changing the experimental conditions of the two reactant beams, as summarized in Supplementary Table 1, different scattering experiments in the collision energy range interested were carried out.

TOF spectra of F/F* + $H_2(v=0, j=1) \rightarrow$ HF + H at different collision energies were accumulated at the corresponding forward scattering direction. As listed in Supplementary Table 1, the data in the collision energy range of 4.0 cm^{-1} to 26.4 cm^{-1} were obtained in two separate experiments. The first experiment is from 13.1 cm^{-1} to 26.4 cm^{-1} and the second experiment is from 4.0 cm^{-1} to 14.6 cm^{-1}. Because the background introduced from discharge F beam source seriously contaminated the TOF spectra near the direction of the F atom beam, the TOF spectra at different scattering angles at the collision energy of 6.8 cm^{-1} were measured by employing an F-atom beam via 248 nm photolysis of the mixture of 5% F_2 / 95% Ne near the nozzle exit. The pulsed valve for producing the F atom beam, the UV photolysis laser, the detection lasers, and the data acquisition system all worked at a repetition rate of 25 Hz. While the pulsed valve for producing the H_2 beam was triggered at 12.5 Hz, allowing the non-reactive background to be recorded and subtracted on a shot-by-shot basis.

The H atom products from the F + $H_2 \rightarrow$ HF + H reaction at the crossing region were detected via a two-step excitation scheme to a highly excited Rydberg state. The H atoms were initially excited from n=1 to n=2 by a 121.6 nm vacuum ultraviolet photon which was generated by the four-wave mixing of 212.5 nm and 845 nm laser in a gas

cell filled with Kr and Ar with a ratio of 1:3. After the VUV excitation, the H atoms in the *n*=2 state were excited to a high Rydberg state with $n \approx 50$ by a 365 nm laser. Both the 121.6 nm and the 365nm laser pulses were temporally and spatially overlapped in the beam crossing region. The neutral Rydberg atoms flew about 256 mm before reaching a fine metal mesh before the MCP detector, and were immediately field ionized after the mesh by the electric field applied between the mesh and the MCP. The ion signals detected by the MCP detector were amplified, discriminated, and then recorded by a 200-MHz multi-channel digital oscilloscope (National Instruments, PXIe-6556). Waveforms obtained by the digital oscilloscope were sent to a field-programmable gate array (FPGA) card (National Instruments, PXIe-7966R FlexRIO FPGA Module). The arrival time of the leading edge of each pulse in the waveforms was extracted by the FPGA card and then recorded by a computer in the form of a time-of-flight (TOF) spectrum.

Table S1. Experimental conditions in the crossed beams experiments

Experiment	Crossing angle /Collision energy	F-atom beam				H_2 molecular beam			
		Gas	Method	Temperature	Velocity	Gas	Temperature	Velocity	
Forward-scattering $F+H_2(v=0, j=1)$	17.5 °- 25 ° @13.1~26.4 cm^{-1}	5% F_2 / 95% He	double-stage discharge	~78 K (electrodes were cooled by liquid N_2)	1.37 km/s	*n*-H_2; *p*-H_2	~78 K (pulsed valve was cooled by liquid N_2)	1.35 km/s	5 k shots per TOF, 20 rounds
Forward-scattering $F+H_2(v=0, j=1)$	15 °- 30 ° @ 4.0~14.6 cm^{-1}	2.5% F_2 /47.5% He /50% Ne	double-stage discharge	~78 K (electrodes were cooled by liquid N_2)	0.87 km/s	80% *n*-H_2 /20% Ne; 80% *p*-H_2 /20% Ne	~78 K (pulsed valve was cooled by liquid N_2)	0.80 km/s	10 k shots per TOF, 12 rounds
DCS of $F+H_2(v=0, j=1)$	22.5 ° at 6.8 cm^{-1}	5% F_2 / 95% Ne	photolysis @ 248 nm	~300 K (pulsed valve was cooled by liquid ethanol)	0.78 km/s	75% *n*-H_2 /25% Ne; 75% *p*-H_2 /25% Ne	~78 K (pulsed valve was cooled by liquid N_2)	0.76 km/s	at scattering angles from -72 ° to 90 ° with an 8 ° interval in the LAB frame, 1700k shots for each TOF spectrum

II. TOF spectra measurement of F + H_2(*v*=0, *j*=1) → HF(*v'*=2, *j'*) + H at the forward scattering direction as a function of collision energy

In order to obtain the TOF spectra in the forward scattering direction of the F + H_2(*v=0, j=1*) → HF (*v'=2, j'*) + H reaction in the collision energy range from 4.0 cm^{-1} to 26.4 cm^{-1}, the data were obtained in two separate experiments. The first experiment is from 13.1 cm^{-1} to 26.4 cm^{-1} and the second experiment is from 4.0 cm^{-1} to 12.3 cm^{-1}, as listed in Supplementary Table 1. The H_2 beam was generated by expanding the H_2 through a pulsed valve (Even-Lavie valve) at a stagnation pressure of 200 psi, which was cooled by liquid nitrogen (~78 K). Two different H_2 samples were used in the experiment: *n*-H_2 (normal H_2) sample, and *p*-H_2 (para H_2) sample. The cooling of the nozzle ensured that nearly all H_2 molecules in the *n*-H_2 molecular beam populated in the *j*=0 and *j*=1 states with a ratio of 25% and 75%. The *p*-H_2 sample was prepared by passing normal H_2 gas through a ferromagnetic catalyst at ~20 K then stored in an aluminum tank at room temperature. After supersonic expansion, nearly all H_2 molecules in the *p*-H_2 molecular beam populated in the *j*=0 state. To achieve lower collision energies, neon was added into the *n*-H_2 and *p*-H_2 samples to obtain a lower speed of H_2 molecules in the molecular beam.

TOF spectra of the forward scattering of the F + H_2(*v=0, j=0,1*) → HF(*v'=2, j'*) + H reaction at different collision energies were obtained for both H_2 samples at the same experimental conditions. To reduce the systematic errors in the experiments, the TOF spectra were measured by switching the *n*-H_2 and *p*-H_2 samples back and forth many times. Since the stagnation pressures of the two different H_2 samples were set to be the same, the density of the *n*-H_2 and *p*-H_2 molecular beams should be the same. Therefore, the TOF spectra (TOFS) of the F + H_2(*v=0, j=1*) → HF(*v'=2, j'*) + H reaction can be obtained by solving the following equations,

$$\text{TOFS}\ (p\text{-}H_2) = \text{TOFS}\ (j=0) \qquad (1)$$

$$\text{TOFS}\ (n\text{-}H_2) = 3/4*\text{TOFS}(j=1) + 1/4*\text{TOFS}(j=0) \qquad (2)$$

$$\text{TOFS}(j=0) = \text{TOFS}\ (p\text{-}H_2) \qquad (3)$$

$$\text{TOFS}(j=1) = 4/3*[\text{TOFS}\ (n\text{-}H_2)-1/4*\text{TOFS}(p\text{-}H_2)] \qquad (4)$$

Typical TOF spectra in the forward scattering at the collision energies of 4.0 and 6.8 cm^{-1} were shown in Supplementary Fig. S2. In the experiment, microchannel plates detectors with a detection area diameter of 20mm were used, and their distance from the scattering center was ~250 mm. Consequently, the angular broadening in the laboratory frame caused by the detector size was about 4.6°, and when this value was converted to the CM frame, the corresponding angular broadening was approximately 5° in the forward direction.

III. Angular resolved DCS measurement of the F + H_2(v=0, j=1) → HF (v'=2, j') + H reaction at the collision energy of 6.8 cm^{-1}

To obtain fully angular resolved DCS of the F+H_2 (v=0, j=1) → HF (v'=2, j') +H reaction at the collision energy of 6.8 cm^{-1}, the TOF spectra from both the F + n-H_2 and the F + p-H_2 were accumulated by the multichannel Rydberg tagging scheme with an angular interval of 8° in the laboratory frame, as described in the previous paragraph. The TOF spectra at different laboratory angles were calibrated using the angular distribution of photodissociation of CH_4 at 121.6 nm, as described in Ref. [37]. The calibrated TOF spectra were converted to the kinetic energy (KE) distribution by a standard Jacobian transformation. The KE distribution of the HF product at a given scattering angle in the LAB frame was fitted by a computer program by adjusting the individual ro-vibrational state intensities of the HF product. By combining ro-vibrational distributions at different scattering directions, ro-vibrational state-resolved differential

cross section (DCSs) was obtained, as shown in Fig. 3.

IV. Estimation of error bars of the experimental data

(1) Estimation on the broadening of the experimental collision energy

In a crossed molecular beams experiment, the collision energy is described by the following equation,

$$E_c = 1/2 * \mu * (v_1^2 + v_2^2 - 2v_1v_2\cos\alpha)$$

Here, μ, v_1, v_2, and α are the reduced mass of the collision system, the velocity of the F atom beam, the velocity of the H_2 molecule beam, and the crossing angle between the two beams, respectively. Therefore, the collision energy broadening is affected by the spread of the crossing angle between the two beams, which both have an angular spread of $\pm 0.5°$, as well as the spreads of the velocities of the two reactant beams.

The velocity distribution of the H_2 beam was measured in this experiment. After turning on both the H_2 molecular beam and the ion gauge in the H_2 beam source chamber, the hot filament of the ion gauge produced H atoms by dissociation of the H_2 molecules which was the main component of the vacuum background in the H_2 beam source chamber. The H atoms generated by the hot filament of the ion gauge and dispersed in the beam source chamber were picked up by the H_2 molecular beam. Therefore, the H atoms were brought into the scattering chamber by the H_2 molecular beam and had the same velocity as the H_2 molecular beam. Using the H atom Rydberg tagging detection, the TOF spectra of the H atom could be obtained. Therefore, the peak velocity and velocity spread of the H atom were determined, from which the peak velocity and velocity broadening of the H_2 molecular beam were obtained. The peak velocity and velocity spread of the F atom beam were obtained by fitting the TOF spectra of the H atom produced from the reactive scattering of $F+H_2$, ensuring the simulated peak width of each rovibrational state matches the experimental one. The

broadening of the crossing angle of the F atom beam and the H_2 molecule beam was estimated based on the distances between the pulsed valve nozzles and the scattering region, which were 112.5 mm and 118 mm, respectively, and the diameter of the scattering region, which was estimated to be ±1.0 mm. The maximum and minimum collision energies can be calculated based on the maximum and minimum velocities of the two molecular beams, as well as the maximum and minimum crossing angles. Finally, the collision energy broadening can be calculated and summarized, as shown in Supplementary Table 2.

(2) Estimation of error bars of the experimental DCS in the forward scattering direction

The experimental TOF spectra (DCSs) were measured at laboratory angles that corresponds to the forward direction of the center-of-mass frame in the collision energies from 4.0 cm^{-1} to 26.4 cm^{-1}, collision energy dependent DCS can thus be determined for the HF(v'=2) product at the forward scattering direction (Supplementary Fig. 2). As shown in Supplementary Table 1, the experimental data was obtained in two separated measurements. The first measurement covering the collision energy range from 13.1 to 26.4 cm^{-1}, and the second measurement covering the collision energy range from 4.0 to 14.6 cm^{-1}. In order to reduce experimental errors, TOF spectra measurement at different collision energies were repeated back and forth many times. The experimental conditions are listed in Supplementary Table 1. For example, in the first measurement covered the collision energies from 13.1 to 26.4 cm^{-1}, TOFs at each collision energy were accumulated back and forth for 20 rounds. In each round, both TOF of F + p-H_2 and F + n-H_2 were accumulated with 5K laser pulses, from which the

TOF of F + H_2(v=0, j=1) was obtained as described in section II. Then the sum of signal counts in each TOF F + H_2(v=0, j=1) was calculated and normalized in each round to evaluate the error bars of one standard deviation (1σ) in the experimental DCSs in the forward scattering direction, as shown in Supplementary Fig. 2.

Table S2. Estimation of the broadening of the experimental collision energy

Collision energy (cm^{-1})	F atom beam		H_2 beam		Crossing Angle			Collision energy broadening (cm^{-1})
	Velocity (km/s)	Velocity spread (km/s)	Velocity (km/s)	Velocity spread (m/s)	Beam crossing angle	F-atom beam angular spread	H_2 beam angular spread	
4.0	0.87	±0.044	0.80	±0.022	15.0°	±0.5°	±0.5°	±0.3
5.3	0.87	±0.044	0.80	±0.022	17.5°	±0.5°	±0.5°	±0.8
6.8	0.87	±0.044	0.80	±0.022	20.0°	±0.5°	±0.5°	±1.0
8.4	0.87	±0.044	0.80	±0.022	22.5°	±0.5°	±0.5°	±1.05
10.3	0.87	±0.044	0.80	±0.022	25.0°	±0.5°	±0.5°	±1.15
12.3	0.87	±0.044	0.80	±0.022	27.5°	±0.5°	±0.5°	±1.2
14.6	0.87	±0.044	0.80	±0.022	30.0°	±0.5°	±0.5°	±1.2
13.1	1.37	±0.070	1.35	±0.017	17.5°	±0.5°	±0.5°	±1.2
17.0	1.37	±0.070	1.35	±0.017	20.0°	±0.5°	±0.5°	±1.25
21.5	1.37	±0.070	1.35	±0.017	22.5°	±0.5°	±0.5°	±1.3
26.4	1.37	±0.070	1.35	±0.017	25.0°	±0.5°	±0.5°	±1.7

V. Quantum dynamical calculations

1) Adiabatic time-independent calculations using ABC code

The adiabatic calculations were performed on the CSZ PES using the time-independent ABC code [36]. The numerical parameters for the adiabatic

calculations are in listed in Supplementary Table 3. Time-independent adiabatic calculation reveals two sharp peaks in the reaction probability of two partial waves: J_{tot}=6, ε=-1 and J_{tot}=7, ε=-1, where ε denotes the total parity of the reaction system (Supplementary Fig. 3). Additionally, a broad peak was observed for the J_{tot}=5, ε=-1 partial wave (Supplementary Fig. 3). The one-dimensional adiabatic effective potentials calculated along the R_{F-HH} direction for selected values of *L*, as shown in Supplementary Fig. 4, indicating that the adiabatic van der Waals well can effectively support quasi-trapped states for *L* = 6 and 7.

Table S3 Numerical Parameters in the calculations using the ABC code.

j_{max}	E_{max} (eV)	k_{max}	J_{tot}	r_{max} (Bohr)	mtr
32	3.2	4	9	70	6000

(2) Diabatic quantum reactive scattering calculations using time-dependent wave packet method

The time-dependent wave packet method used in the current work has been described in details in previous works [16][43]. A brief description is provided here. The diabatic quantum dynamics calculations were performed based on the theoretical model developed by Alexander and co-workers [44], which fully incorporate the open-shell characteristics and spin-orbit effects of the F atom. Following their approach, we adopt a Hund's case (a) representation for the present calculations, as within this theoretical framework it offers a more appropriate description in the interaction region, where the electronic angular momentum is strongly coupled to the internuclear axis. There are six electronic states in the F + H_2 reaction due to the three spatial orientations of the 3*p* hole

on the F atom and two possible spin-projection quantum numbers. The Hamiltonian in Jacobi coordinate for the six-state model could be written as [45]

$$\hat{\mathbf{H}} = -\frac{1}{2\mu_R}\frac{\partial^2}{\partial R^2} + \frac{1}{2\mu_r}\frac{\partial^2}{\partial r^2} + \frac{\mathbf{L}^2}{2\mu_R R^2} + \frac{\mathbf{j}^2}{2\mu_r r^2} + \mathbf{V} \#$$

Here, R is the length of the vector $\vec{R}$ from F atom to the H_2 center of mass, and r is the H_2 bond length. μ_R is the reduced mass between the center of mass of F and H_2 molecule. μ_r is the reduced mass of H_2. **L** is the orbital angular momentum operator, and **j** is the rotational angular momentum operator of H_2. $\mathbf{V}$ is the potential energy operator, including nonadiabatic coupling terms $\mathbf{V}_{el}$ and electrostatic spin-orbit coupling term $\mathbf{V}_{so}$. In an *ab initio* calculation on the FH_2 system, the wave function is expanded in a set of Cartesian molecular orbitals. The results are three electronically adiabatic PESs for the three states, two of A' and one of A'' reflection symmetry. These three adiabatic PESs are then transformed into four quasi-diabatic PESs, designated V_{zz}, V_{xx}, V_{yy}, and V_{xz}. In terms of these, the potentials are defined by $V_\Sigma = V_{zz}, V_\Pi = \left(V_{yy} + V_{xx}\right)/2, V_2 = \left(V_{yy} - V_{xx}\right)/2$, and $V_1 = V_{xz}/\sqrt{2}$. The electronic degrees of freedom can be represented as

$$|\lambda\,\sigma\rangle \equiv |l\lambda\,\rangle|s\sigma\,\rangle \#$$

Here, λ and σ represent the projections of the electron orbital angular momentum *l* and spin angular momentum *s*, respectively, both of which are assumed to align along the Jacobi vector $\vec{R}$. The nonadiabatic coupling terms $\mathbf{V}_{el}$ is written as

$$
\begin{array}{ccccccc}
 & |\Sigma\rangle & |\bar{\Sigma}\rangle & |\Pi_1\rangle & |\bar{\Pi}_1\rangle & |\Pi_{-1}\rangle & |\bar{\Pi}_{-1}\rangle \\
 & k_a = \frac{1}{2} & -\frac{1}{2} & \frac{3}{2} & \frac{1}{2} & -\frac{1}{2} & -\frac{3}{2} \\
|\Sigma\rangle & V_\Sigma & 0 & -V_1 & 0 & V_1 & 0 \\
|\bar{\Sigma}\rangle & 0 & V_\Sigma & 0 & -V_1 & 0 & V_1 \\
|\Pi_1\rangle & -V_1 & 0 & V_\Pi & 0 & V_2 & 0 \\
|\bar{\Pi}_1\rangle & 0 & -V_1 & 0 & V_\Pi & 0 & V_2 \\
|\Pi_{-1}\rangle & V_1 & 0 & V_2 & 0 & V_\Pi & 0 \\
|\bar{\Pi}_{-1}\rangle & 0 & V_1 & 0 & V_2 & 0 & V_\Pi
\end{array}
$$

where k_a is the summation of λ and σ. The electrostatic spin-orbit coupling term $\mathbf{V}_{so}$ is written as

$$
\begin{array}{ccccccc}
 & |\Sigma\rangle & |\bar{\Sigma}\rangle & |\Pi_1\rangle & |\bar{\Pi}_1\rangle & |\Pi_{-1}\rangle & |\bar{\Pi}_{-1}\rangle \\
 & k_a = \frac{1}{2} & -\frac{1}{2} & \frac{3}{2} & \frac{1}{2} & -\frac{1}{2} & -\frac{3}{2} \\
|\Sigma\rangle & 0 & 0 & 0 & -2^{\frac{1}{2}}B & 0 & 0 \\
|\bar{\Sigma}\rangle & 0 & 0 & 0 & 0 & -2^{\frac{1}{2}}B & 0 \\
|\Pi_1\rangle & 0 & 0 & -A & 0 & 0 & 0 \\
|\bar{\Pi}_1\rangle & -2^{\frac{1}{2}}B & 0 & 0 & A & 0 & 0 \\
|\Pi_{-1}\rangle & 0 & -2^{\frac{1}{2}}B & 0 & 0 & A & 0 \\
|\bar{\Pi}_{-1}\rangle & 0 & 0 & 0 & 0 & 0 & -A
\end{array}
.
$$

where

$$A(R,r,\theta) \equiv i\langle \Pi_y | H_{\mathrm{so}} | \Pi_x \rangle \#$$

and

$$B(R,r,\theta) \equiv \langle \bar{\Pi}_x | H_{\mathrm{so}} | \Sigma \rangle . \#$$

The six diabatic potential energy surfaces (PESs) used in our current calculations are based on the iCSZ-LWAL PESs used in previous works [16][20][37][46] with a slight improvement made to the electrostatic spin-orbit coupling term.

The total angular basis function for the six-state model is written as

$$|JMK\rangle|jk\rangle|\lambda\sigma\rangle = \left(\frac{2J+1}{8\pi^2}\right)^{\frac{1}{2}} D^{J*}_{MK}(\boldsymbol{\Omega}) Y_{jk}(\theta) |l\lambda\rangle |s\sigma\rangle \#$$

Here, $|jk\rangle$ is the rotational wave function of the diatomic molecule, where k is the projection of the diatomic rotational angular momentum along the Jacobi vector $\vec{R}$.

D^J_{KM} is Wigner rotation matrix element, where the three Euler angles Ω define the space-frame orientation and J designates the total angular momentum (nuclear rotational plus electronic) with spaceframe projection M and body-frame projection K. The reduced rotation matrix element $Y_{jk}(\theta)$ describes angular motion of the diatomic molecule. We use definite-K linear combinations,

$$|JMKjk\lambda\sigma\varepsilon\rangle = 2^{-1/2}[|JMK\rangle|jk\rangle|\lambda\sigma\rangle$$

to reduce the computational effort since there will be no coupling between states of different values of ε. For these body-frame functions, we adopt the convention used by molecular spectroscopists, defining parity based on the overall symmetry with respect to reflection in the triatomic plane. The parity is $+(-1)^{J-s}$ when $\varepsilon = +1$ and $-(-1)^{J-s}$ when $\varepsilon = -1$. The total wave function is expanded as

$$\Psi^{JM\varepsilon}(\vec{R},\vec{r},t) = \sum_{jKk\lambda\sigma} \psi^{jKk\lambda\sigma\varepsilon}(R,r,t)|JMKjk\lambda\sigma\varepsilon\rangle\#$$

Although the summation formally includes the projection quantum numbers k, λ and σ, their values are constrained by the requirement $K = \lambda + \sigma + k,$ and the total projection quantum number K must be positive, consistent with the definite-K basis expansion.

The initial wave packet is constructed in a fully coupled space-frame basis. The space-frame electronic wave functions $|lm_l\rangle$ and $|sm_s\rangle$ of the F atoms are coupled to yield the total atomic orbital angular momentum j_a. This is then coupled with the space-frame wave function for the rotational motion of the H_2 molecule $|jm_j\rangle$, yielding the total internal angular momentum j_{12}. Finally, j_{12} is coupled with the orbital motion of the triatomic complex $|LM_L\rangle$ to yield the total angular momentum J. We refer to these space-frame functions as $|(ls)j_a j j_{12} LJM\rangle$. The parity of these

space-frame functions is given by:

$$\mathbf{i}_{sp}|(ls)j_a j j_{12} LJM\rangle = (-1)^{L+l+j}|(ls)j_a j j_{12} LJM\rangle \#$$

We apply the reactant-product decoupling (RPD) method [46] to obtain state-to-state information. The parameters used in the calculations for the six-state model are listed in Supplementary Tables 4 and 5. Further theoretical details on the six-state model can be found in ref. [44] and [45].

Figure S5 show the comparison between the forward-scattering DCSs calculated using the adiabatic and diabatic models. Figure S6 presents the relative reaction probabilities for selected spin-orbit fine-structure partial waves, identifying the dominant partial wave contributions to the forward-scattering peak. Figure S7 illustrates the integral cross sections (ICSs) from the diabatic models, together with the *L*- and *J*-resolved partial-wave contributions to the ICSs (panels A and B, respectively).

3) The one-dimensional effective potentials for the incident adiabatic states

Since the collision energy considered in this work falls within the cold regime, when the distance between F and H_2 is sufficiently large (R > 5 Bohr), the interaction between the colliding particles becomes too weak to alter the ro-vibrational state of the molecule. The one-dimensional effective potential can be obtained by considering only the combined effects of the potential energy and centrifugal interaction. Consequently, the system's Hamiltonian can be simplified to:

$$\hat{\mathbf{H}}' = \frac{\mathbf{L}^2}{2\mu_R R^2} + \mathbf{V}$$

The explicit form of the potential energy operator $\mathbf{V}$ has been discussed earlier. The

centrifugal interactions in the reactant channel are expressed as:

$$
\begin{aligned}
\mathbf{L}^2 | Kk\lambda\sigma\rangle &= (J - l - s - j)^2 | Kk\lambda\sigma\rangle \\
&= [J(J+1) + j(j+1) + \langle l^2\rangle + 3/4 - 2K\lambda - 2K\sigma - 2Kk + 2\lambda\sigma + 2k\lambda + 2k\sigma] | Kk\lambda\sigma\rangle \\
&\quad - \xi_{JK}^{\pm}\xi_{jk}^{\pm} | K \pm 1, k \pm 1 \lambda, \sigma\rangle \\
&\quad - \xi_{JK}^{\pm}\alpha_{\pm} | K \pm 1, k \lambda \pm 1, \sigma\rangle \\
&\quad - \xi_{JK}^{\pm}\left[3/4 - \sigma(\sigma \pm 1)\right]^{1/2} | K \pm 1, k, \lambda \sigma \pm 1\rangle \\
&\quad + \xi_{jk}^{\pm}\alpha_{\mp} | K, k \pm 1 \lambda \mp 1, \sigma\rangle \\
&\quad + \xi_{jk}^{\pm}\left[3/4 - \sigma(\sigma \mp 1)\right]^{1/2} | K, k \pm 1 \lambda, \sigma \mp 1\rangle \\
&\quad + \alpha_{\mp}\left[3/4 - \sigma(\sigma \pm 1)\right]^{1/2} | K, k \lambda \mp 1, \sigma \pm 1\rangle
\end{aligned}
$$

where $\xi_{xy}^{\pm} = [x\ (x + 1) - y\ (y\ \pm 1)]^{1/2}$. For a given total angular momentum and parity, the effective potential for the adiabatic incident states at a given value of R can be obtained by diagonalizing the matrix representation of the Hamiltonian operator acting on the total angular basis functions $|JMK\rangle|jk\rangle|\lambda\sigma\rangle$. The resulting eigenvalues correspond to the effective potential of the adiabatic states, while the eigenvectors can be utilized to project the resonance wavefunctions onto these adiabatic states, as illustrated in Supplementary Fig. 7.

Table S4 Numerical parameters for reactant Jacobi coordinate propagation, six-state model. (atomic unit was used unless otherwise stated)

Grid/basis range and size	$R \in [0.0, 200.0], N_R = 2047$ (among them 127 for interaction region and 255 for asymptotic interaction)
	$r \in [0.6, 13.0], N_r = 130$
	$j_{\min} = 0 \sim j_{\max} = 100, N_j = 51$ over $[0, 90°]$
Absorption functions	$\exp[-0.08 \times \Delta_t (r - 11.0)^2]$ for $11.0 \le r \le 13.0$
	$\exp[-0.00003 \times \Delta_t (R - 25.0)^2]$ for $25.0 \le R \le 200.0$

Initial wavepacket $\exp\left[-\frac{(R-R_0)^2}{2\Delta_R^2} ik_0R\right]$	
	$R_0 = 18.0$
	$\Delta_R = 2.0$
	$k_0 = (2E_0\mu_R)^{1/2}$ with $E_0 = 0.005$ eV
Total propagation time	1500000
Time step	10

Table S5 Numerical parameters for product Jacobi coordinate propagation, six-state model.

Grid/basis range and size	$R \in [2.3, 42.3], N_R = 323$
	$r \in [0.5, 5.0], N_r = 12$
	$j_{\min} = 0 \sim j_{\max} = 30, N_j = 31$ over $[0, 180°]$
Absorption functions	$\exp[-0.002 \times \Delta_t(R-32.3)^2]$ for $32.3 \le R \le 42.3$
Total propagation time	2500000
Time step	10
Position where the scattering wave function was calculated	$R^{\infty} = 30.0$

Supplementary Figures & Captions

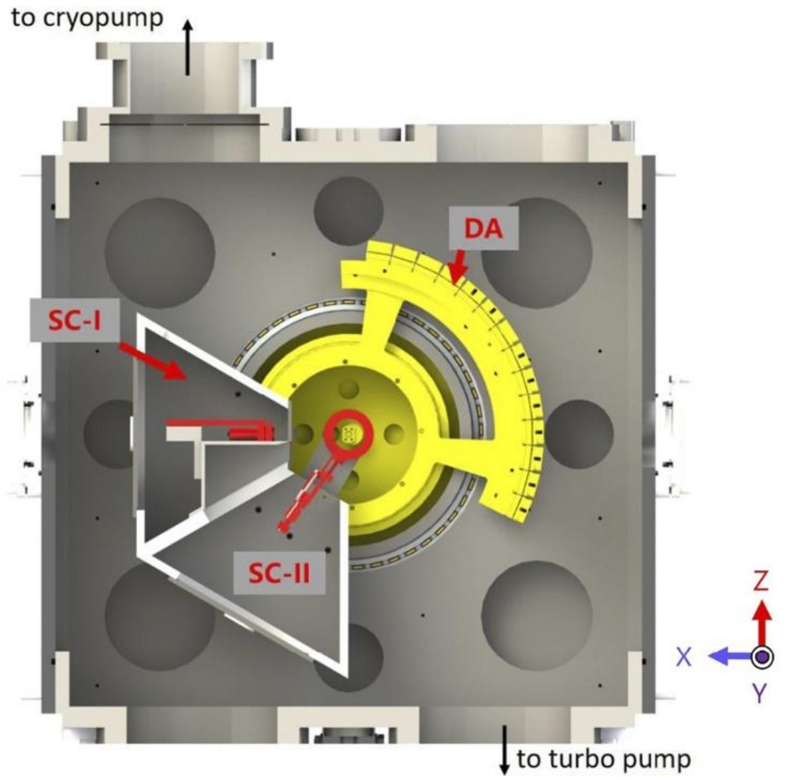

Fig. S1 The schematic diagram of the experimental device used in this study. SC-I: source chamber I, SC-II: source chamber II, and DA: detector assembly. The detection lasers propagate perpendicular to the XZ plane, and the polarization of the 845 nm laser is parallel to the X-axis.

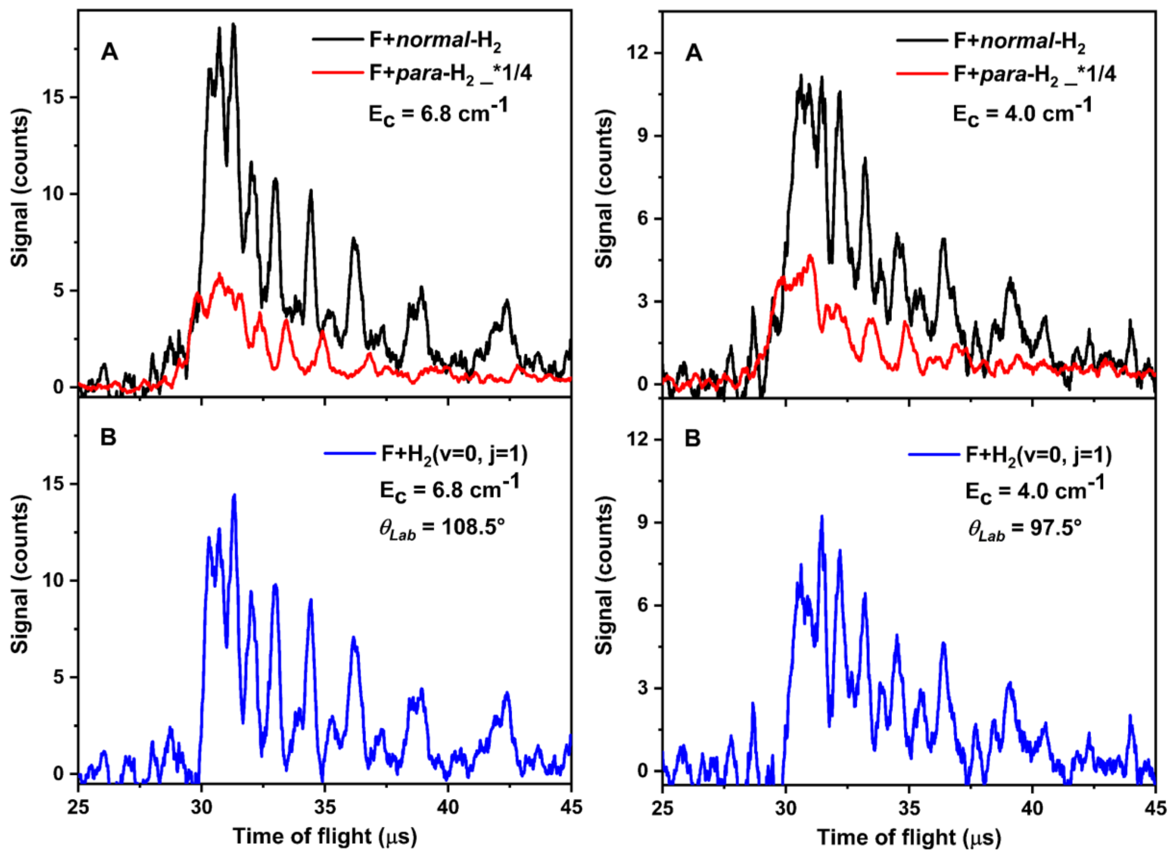

Fig. S2 Time-of-flight spectra of the H atom products from the F+n-H_2 and F+p-H_2 (upper panel), and F+H_2($v=0$, $j=1$) (B) reaction at collision energies of 6.8 cm^{-1} (left panel) and 4.0 cm^{-1} (right panel).

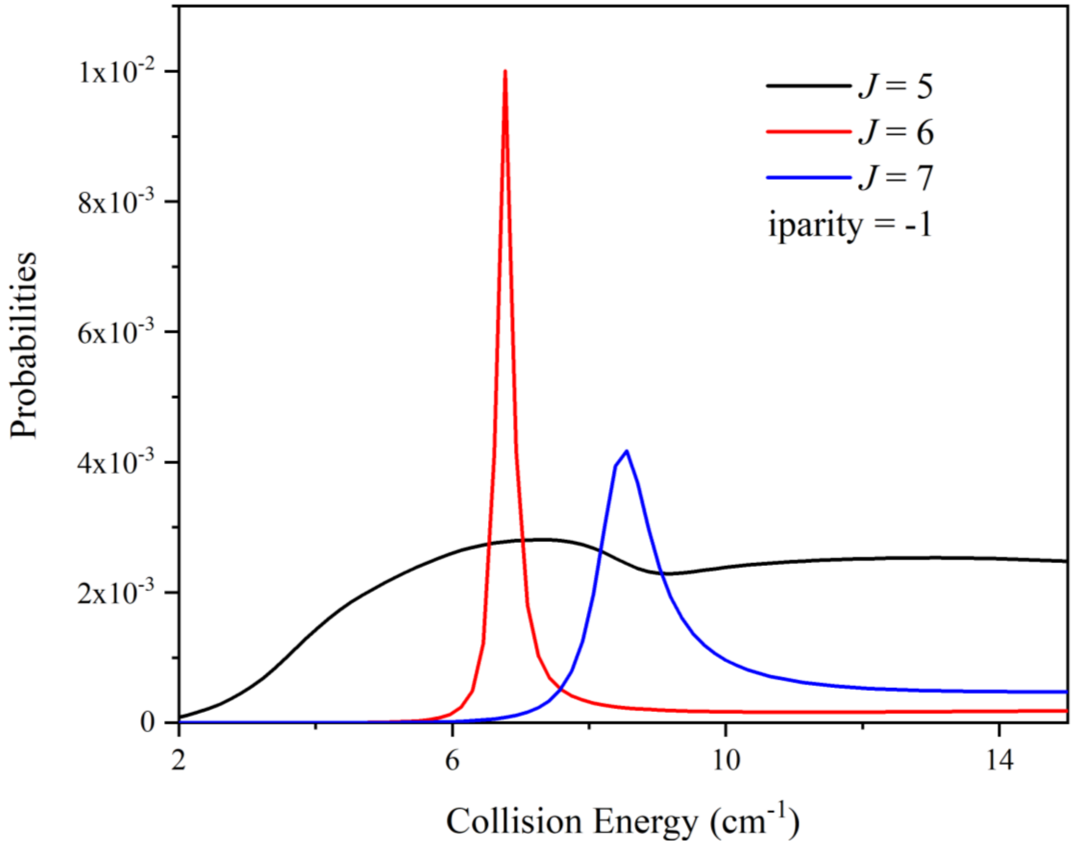

Fig. S3 The reaction probabilities of the J_{tot}=5/6/7, ε=-1 partial waves. These three partial waves contribute to the resonance peak structure of the adiabatic QSSFSS spectra.

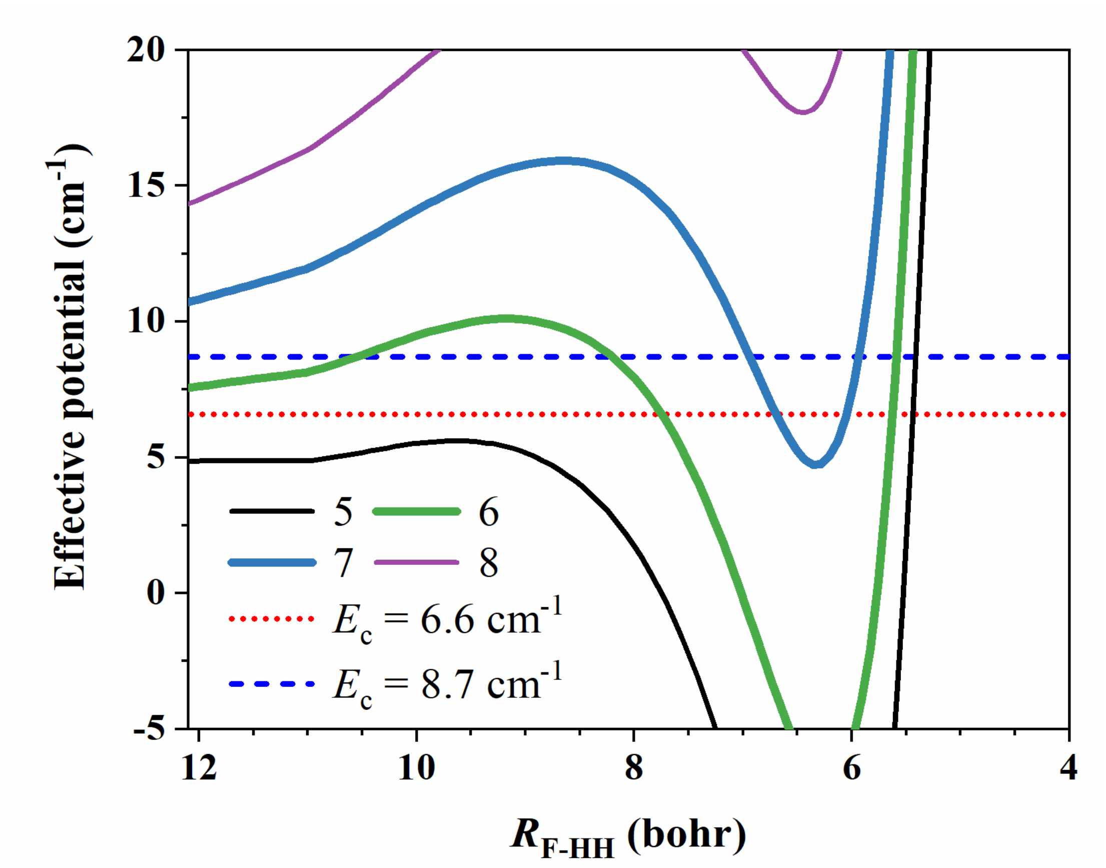

Fig. S4 One-dimensional adiabatic effective potentials on the $R_{F\text{-}HH}$ direction, calculated in the adiabatic model for H_2 in the (v=0, j=1) state and selected values of L.

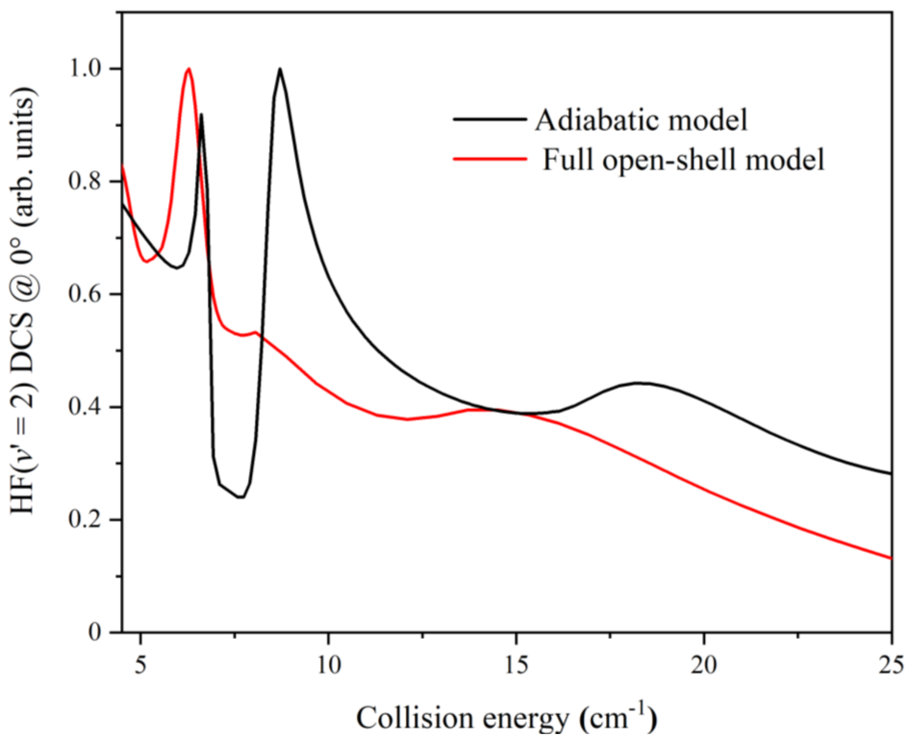

Fig. S5 Forward-scattering DCS calculated using the adiabatic and full open-shell model as a function of collision energy.

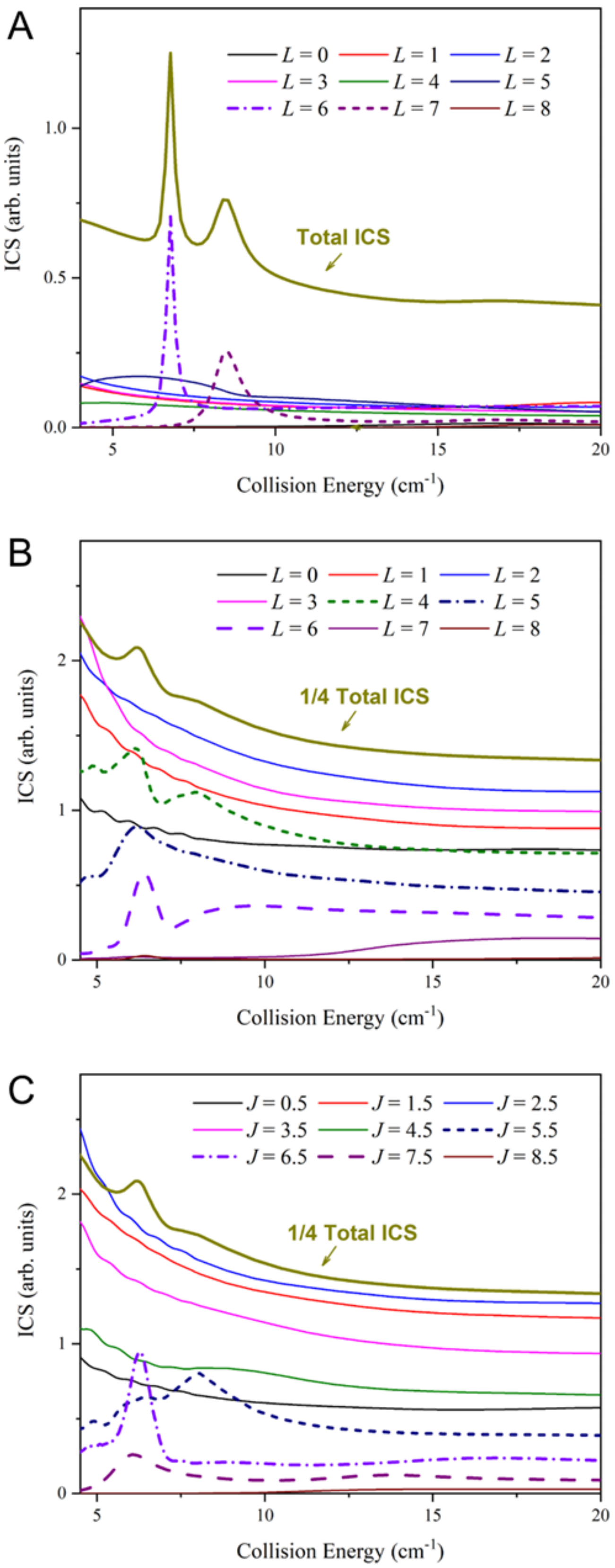

Fig. S6 Integral cross sections and partial wave contributions for the F($^2P_{3/2}$)+H_2(v=0, j=1) reaction in the low collision energy regime. Adiabatic results: ICSs and L-resolved partial-wave contributions. (B, C) Diabatic results: ICSs with partial-wave contributions resolved by L (B) and by J (C).

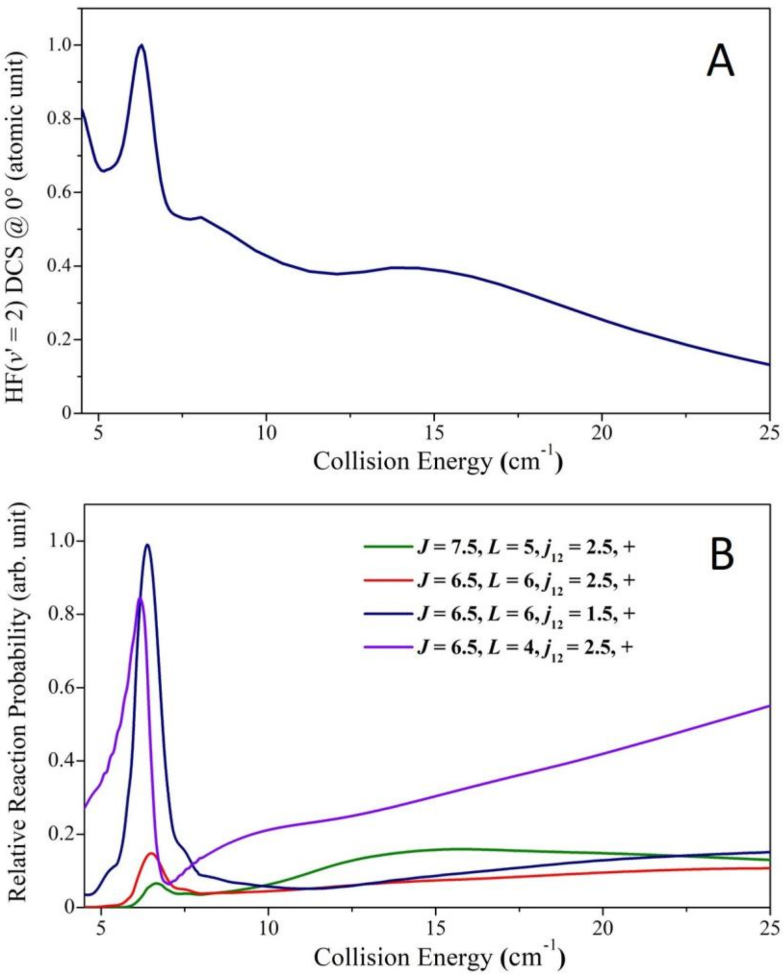

Fig. S7 Reaction probabilities for the $F(^2P_{3/2})+H_2(v=0,\ j=1)\rightarrow HF\ (v'=2,\ j')$ +H reaction in the low collision energy regime. (**A**) Forward-scattering DCS calculated using the full open-shell model as a function of collision energy. (**B**) The relative reaction probability, scaled by the statistical factor (2*J*+1) for selected spin-orbit fine-structure partial waves, is shown as a function of collision energy.

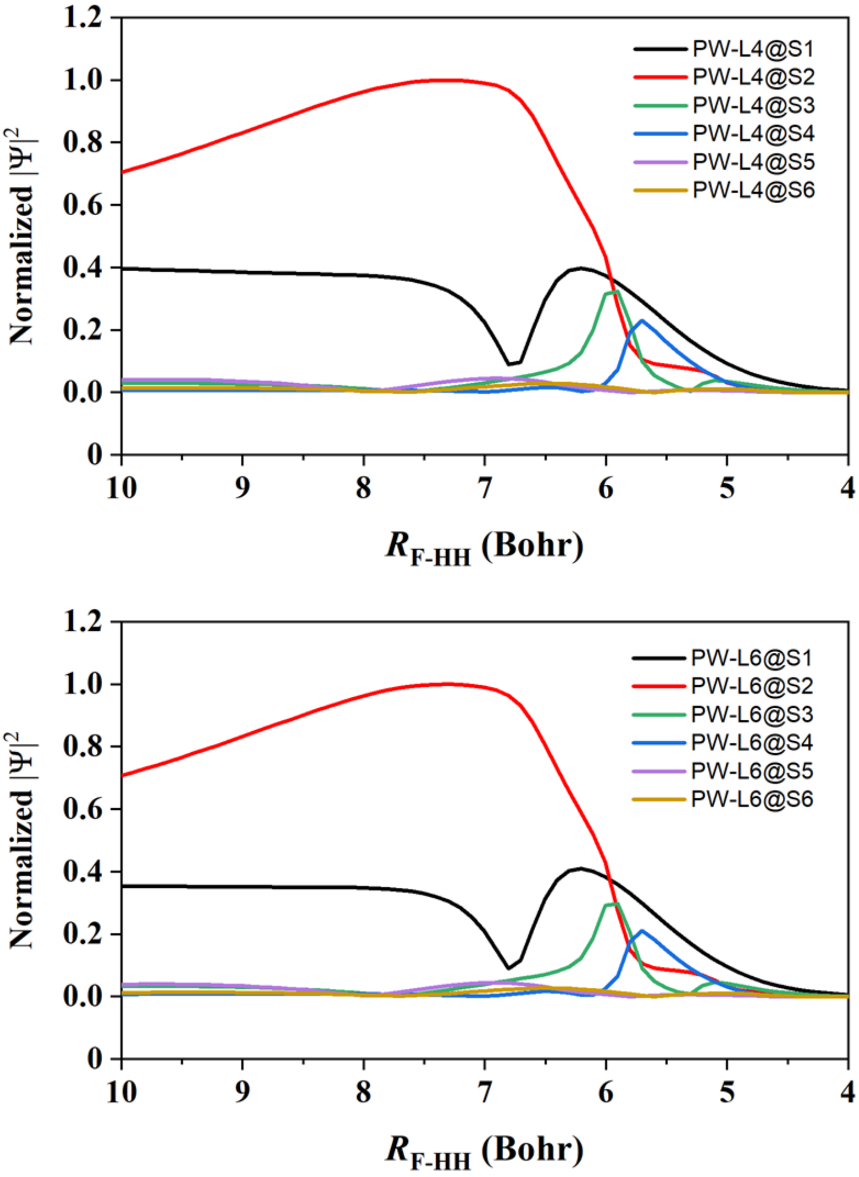

Fig. S8 Resonance wave functions distribution on the S1-S6 states for PW-L4 (upper panel) and PW-L6 (lower panel).

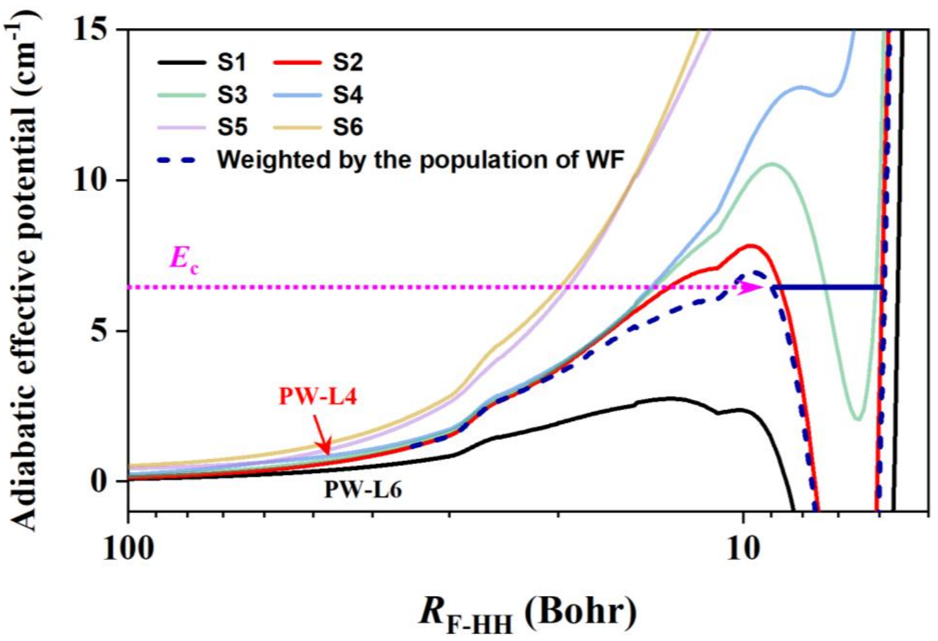

Fig. S9 Extended version of Fig. 4C along the *R* coordinate. The blue line represents the one-dimensional adiabatic effective potentials obtained by averaging over the potential, weighted by the population of the time-independent scattering wave functions.

References

40. Ren ZF, Qiu MH, Che L *et al.* A double-stage pulsed discharge fluorine atom beam source. *Rev Sci Instrum* 2006; **77**: 016102.

41. Wang YF, Du TY, Dai DX *et al.* A slow and clean fluorine atom beam source based on ultraviolet laser photolysis. *Chin J Chem Phys* 2021; **34**: 381–385.

42. Huang JY, Liu S, Zhang DH. Time-dependent wave packet dynamics calculations of cross sections for ultracold scattering of molecules. *Phys Rev Lett* 2018; **124**: 143404.

43. Alexander MH, Manolopoulos DE, Werner H-J. An investigation of the F+H2 reaction based on a full ab initio description of the open-shell character of the F(2P) atom. *J Chem Phys* 2000; **113**: 11084–11100.

44. Sun ZG, Zhang DH, Alexander MH. Time-dependent wavepacket investigation of state-to-state reactive scattering of Cl with para-H_2 including the open-shell character of the Cl atom. *J Chem Phys* 2010; **132**: 034308.

45. Lique F, Li G, Werner HJ *et al.* Communication: non-adiabatic coupling and resonances in the F + H2 reaction at low energies. *J Chem Phys* 2011; **134**: 231101.

46. Peng T, Zhang JZH. A reactant-product decoupling method for state-to-state reactive scattering. *J Chem Phys* 1996; **105**: 6072–6074.
